# 

**Published:** 1882-01

**Authors:** 


### Pamphlets.

Occlusion of the Gravid Uterus. By Joseph A. Eve, M. D., Augusta, Ga. Reprinted from Vol. V. Gynecological Transactions.

Reform in medical education the aim of the Academy. Annual address delivered before the American Academy of Medicine, at New York, September 20, 1881. By Edward T. Caswell, A. M., M. D., president.

Education in Charleston, S. C The disabilities of the unaided south in public school facilities. Extract from the annual review of the mayor of Charleston.

Rudolph Virchow. An address introductory to the course of lectures of the term of 1881–82 of the College of Physicians and Surgeons, New York. By A. Jacobi, M. D. Reprinted from the New York Medical Record.

Observations on the origin, character and treatment of oinomania. By T. L. Wright, M.D., Bellefontaine, Ohio. Reprinted from the Alienist and Neurologist.

Historical sketch of the medical societies of Baltimore, Maryland, from 1730 to 1880. By G. Lane Tannyhill, M. D. Reprinted from the transactions of the faculty, 1881.

Contributions to the study of the toxicology of cardiac depressants. I. Carbolic Acid; A summary of fifty-six cases of poisoning, with a study of its physiological action. By Edward T. Reichert, M. D. Reprinted from the American Journal of the Medical Sciences.

